# Supplementary material for: On the Origin of Neo-Sex Chromosomes in the Neotropical Dragonflies Rhionaeschna bonariensis and R. planaltica (Aeshnidae, Odonata)
Source: Insects. 2022 Dec 15;13(12):1159. doi: 10.3390/insects13121159 (PMC9784284; doi:10.3390/insects13121159)
Supplement: Supplementary file 1 [file insects-13-01159-s001.zip › insects-2063340-supplementary.pdf]

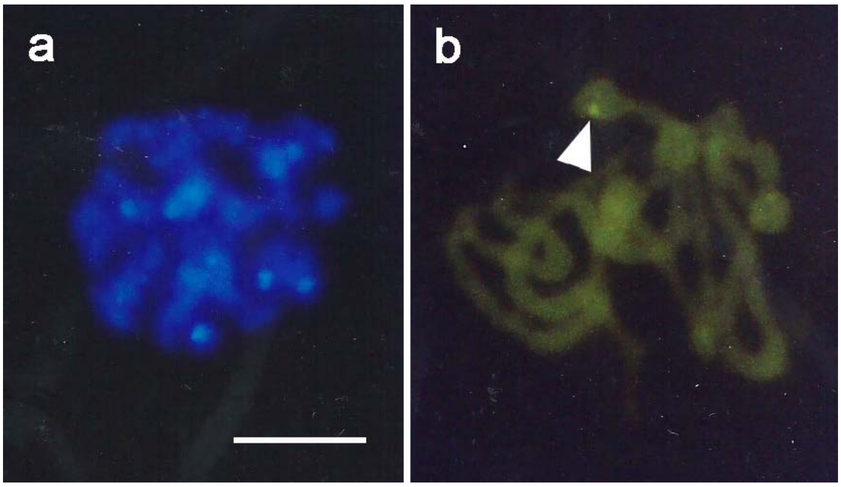

Figure S1. DAPI-CMA_3 banding of male meiotic chromosomes in *Rhionaeschna planaltica*. **a** - DAPI banding, diplotene. **b** - CMA_3 banding, pachytene; arrowhead points to CMA_3-positive band. Bar = 10 μm.
